# Correction to: Sepsis increases perioperative metastases in a murine model

**DOI:** 10.1186/s12885-018-4248-2

**Published:** 2018-04-17

**Authors:** Lee-Hwa Tai, Abhirami A. Ananth, Rashmi Seth, Almohanad Alkayyal, Jiqing Zhang, Christiano Tanese de Souza, Phillip Staibano, Michael A. Kennedy, Rebecca C. Auer

**Affiliations:** 1 Deparment of Biochemistry, Microbiology, and Immunology, Faculty of Medicine, University of Ottawa, Ottawa, Canada; 2 Center for Innovative Cancer Research, Ottawa Hospital Research Institute, Ottawa, Canada; 3 Department of Surgery, Division of General Surgery, University of Ottawa, Ottawa, Canada; 4 Department of Medical Laboratory Technology, University of Tabuk, Tabuk, Saudi Arabia; 5 Department of Neurosurgery, The Second Hospital of Shandong University, Shandong, China; 6 Department of Cellular and Molecular Medicine, Faculty of Medicine, University of Ottawa, Ottawa, Canada; 7 Ottawa General Hospital, 501 Smyth Road, 1617 CCW, Box 134, Ottawa, ON K1H8L6 Canada

## Correction

It has been highlighted that the original manuscript [1] contains a typesetting error in Fig. 1 and the Fig. 1c panel has been inadvertently duplicated in panel Fig. 1d. This does not affect the results and conclusions of the article. The correct version of Fig. 1 is included with this Correction. The original article has been updated.

**Fig. 1:**
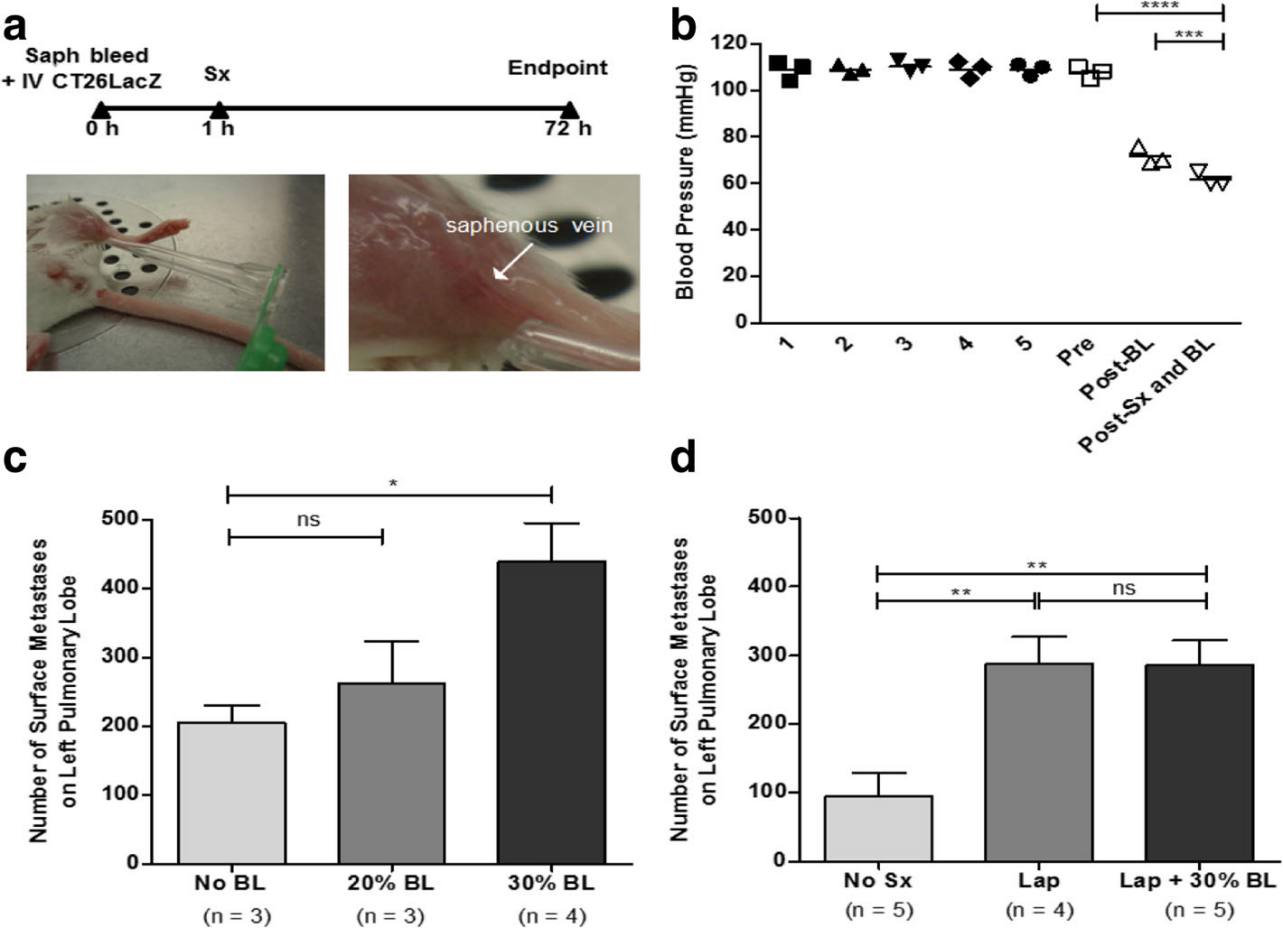
Hemorrhagic shock does not increase metastatic disease. **a** *Experimental overview.* BALB/c mice were bled through the saphenous vein (indicated by the white arrow) and subsequently injected intravenously (IV) through the tail vein with 3 × 10^5^ CT26LacZ cells. Approximately 1 h later, surgical stress (sx) was generated by laparotomy (Lap) (5 cm incision). Mice were sacrificed at 72 h to quantify lung metastases. **b** *Blood pressure is reduced following surgical stress and blood loss.* Blood pressure (mmHg) was measured following a 5-day training period (Day 1–5), prior to bleeding (Pre), immediately following bleeding (Post-BL), and immediately following surgical stress (Post-Sx and BL, *n* = 3). **c** *Blood loss increases metastatic burden.* Lung metastases were measured on Day 3 following no blood loss (no BL, *n* = 3) or 20% (20% BL, *n* = 3) or 30% blood loss (30% BL, *n* = 4). **d** *Blood loss does not increase metastatic disease in conjunction with surgical stress.* Lung metastases were measure on Day 3 in mice that did not undergo surgical stress (No Sx, *n* = 5) and animals undergoing a laparotomy (Lap, *n* = 4) alone or in combination with 30% blood loss (Lap + 30% BL, *n* = 5)**.** Error bars represent ± SEM
